# Tuberculosis-related serpiginous choroiditis: aggressive therapy with dual concomitant combination of multiple anti-tubercular and multiple immunosuppressive agents is needed to halt the progression of the disease

**DOI:** 10.1186/s12348-022-00282-6

**Published:** 2022-02-08

**Authors:** Ioannis Papasavvas, Bruno Jeannin, Carl P. Herbort

**Affiliations:** Retinal and Inflammatory Eye Diseases, Centre for Ophthalmic Specialized Care (COS) & Clinic Montchoisi, Rue Charles-Monnard 6, 1003 Lausanne, Switzerland

**Keywords:** Serpiginous choroiditis, Serpiginous-like choroiditis, Multifocal choroiditis, Tuberculosis, Indocyanine green angiography, Blue light fundus autofluorescence, Immune-mediated choriocapillaritis

## Abstract

**Background/purpose:**

Serpiginous-like choroiditis is a rare immune-mediated sub-entity of tubercular uveitis with a usually deleterious outcome. Treatment is still controversial. The purpose in this case series is to indicate that only aggressive treatment comprising multiple anti-tubercular and multiple immunosuppressive agents seems to be able to halt the disease progression.

**Methods:**

This retrospective case series included patients diagnosed with Interferon Gamma Release Assays (IGRA) -positive serpiginous choroiditis, seen at the Centre for Ophthalmic Specialized Care, Lausanne, Switzerland, treated with combined multiple antitubercular and immunosuppressive agents at presentation and having a sufficient follow-up. Disease history before referral, appraisal of disease, treatment modalities and follow-up were analyzed. Inclusion criteria were positive IGRA patients with serpiginous choroiditis with complete Spectral-Domain Optic coherence tomography (SD-OCT) and angiography images.

**Results:**

From 2001 to 2020, 24 of 1525 new patients (0.26%) were diagnosed as serpiginous choroiditis. 10/24 were related to tuberculosis (positive IGRA and/or hyper-positive Mantoux test), 8/24 were IGRA negative and in 6 there was no information available. 4/10 tuberculosis related serpiginous patients fulfilled the inclusion criteria. Mean age was 39 ± 5.3 years. Snellen best corrected vision acuity (BCVA) at presentation in 3/4 where the macula was preserved was 0.96 ± 0.08. In 3/4 patients, treatment with multiple tuberculostatic therapy combined with multiple immunosuppressive agents, started at presentation or in the initial months after the first consultation, was shown to stop the progression of the disease, with a retained visual acuity of 1.0. One patient with macular involvement and a bilateral visual acuity of hand movements after 11 years of insufficient treatment, improved his visual acuity to 0.25 OD and 0.05 OS and presented a substantial visual field improvement that stabilized once multiple anti-tubercular and immunosuppressive therapy was introduced.

**Conclusion:**

IGRA-positive serpiginous choroiditis (serpiginous-like choroiditis) could be halted by combined multiple tuberculostatic and multiple immunosuppressive agents, as seen in our study where 3/4 early treated patients had conserved central function and one late treated patient had recovered a substantial amount of visual field. In all 4 patients this treatment regimen halted the progression of the disease.

## Introduction

Serpiginous choroiditis (SC) is a immune-mediated, bilateral asymmetrically evolving disease of the choriocapillaris involving secondarily the retinal pigment epithelium (RPE), the outer retina and finally the whole chorio-retina, leaving full-thickness scars [1]. The disease has a recurrent course with alternating quiet and progressing phases. It is situated on the more severe side of the group of inflammatory choriocapillaropathies [2]. Classically SC was considered a disease driven by an autoimmune response following an unknown viral trigger factor [3]. At the beginning of the twenty-first century the role of tuberculosis (TB) in the development of SC in a substantial number of cases in Indian series was reported [4, 5]. The hypothesis of the pathogenesis is an autoimmune response stimulated by *Mycobacterium tuberculosis*. The entity resembles SC’s serpentine progression but does not arise from the disc and it is frequently associated with moderate vitritis (absent in classic SC). Different denominations have been used to describe presumed TB-related SC such as serpiginoïd choroiditis, serpiginous-like choroiditis, multifocal serpiginoïd choroiditis [1]. More recently, several reports showed that *Mycobacterium tuberculosis* may also be involved in the development of SC-like choroiditis in non-endemic areas [6]. Consequently, possible involvement of tuberculosis is routinely investigated by performing Interferon-γ release assays (IGRAs) to detect memory T cells sensitized to *Mycobacterium tuberculosis* antigens [7].

There are different presentations of the disease such as a placoid form with a yellowish lesion that occupy the posterior pole. Another presentation described is a multifocal serpiginous form characterized by multiple yellow-whitish lesions in posterior pole and mid periphery corresponding to areas of choriocapillaritis [8].

Multimodal imaging has allowed to better characterize the evolution of the disease. New imaging techniques such as optical coherence tomography angiography (OCT-A) [9] and enhanced depth imaging OCT (EDI-OCT) [10] are helpful in the follow up of serpiginous-like choroiditis. As the disease involves principally the choriocapillaris, OCT-A was shown to account for the evolution of the disease [11–13]. However, OCT-A only shows a limited area of the posterior pole and for global survey of the lesions, ICGA still represents the gold standard exam for diagnosis and follow up as it can better visualize the choriocapillaris and as result allows a better follow up of a choriocapillaritis such as serpiginous-like choroiditis with a global view [14]. Blue light fundus autofluorescence (BL-FAF) can also yield useful information to follow and monitor serpiginous like choroiditis [10] as it is not invasive and can demonstrate the evolution of the pathology.

As the ideal treatment is still under consideration we would like to demonstrate, with our study, the benefit of a dual multiple immunosuppressive and multiple anti-tubercular treatment in order to stop the evolution of the disease which can otherwise be deleterious for the eye.

## Methods

This retrospective study of a case series was performed in the Centre for Ophthalmic Specialised care (COS), Lausanne, Switzerland. Patients diagnosed from 2001 to 2020 with serpiginous choroiditis with a positive IGRA test and who were treated concomitantly with dual multiple immunosuppressives, and multiple anti-TB agents were included. Poor quality of images or insufficient follow up were the exclusion criteria. Imaging analysis included spectral domain optical coherence tomography (SD-OCT) and enhanced depth imaging OCT (EDI-OCT) (Heidelberg Engineering GmbH, Heidelberg, Germany), OCT angiography (OCT-A) (AngioVue®, Optovue, Fremont, CA, USA) Fluorescein and Indocyanine angiography (FA, ICGA) (Heidelberg Engineering GmbH, Heidelberg, Germany) before and after the instauration of the treatment. Best corrected visual acuity (BCVA), intraocular pressure (IOP) and routine ocular examination, as well as laser flare photometry (LFP) were performed at presentation and during the follow up of patients. Anti-TB treatment was either triple or quadruple therapy including Isoniazid, Rifampicin, Pyrazinamide, and Ethambutol for 2 months followed by Isoniazid and Rifampicin for at least 8 months. Immunosuppression comprised triple therapy among the following agents: prednisone, cyclosporine, azathioprine, mycophenolic acid and infliximab.

## Results

From 2001 to 2020, 24 of 1525 new patients (0.26%) were diagnosed as serpiginous choroiditis (SC). 10/24 (41.6%) were related to tuberculosis as they had a positive IGRA test and/or hyper-positive Mantoux test. 8/24 (33%) were IGRA negative and in 6 cases (25%) there was no information available. Four out of 10 (40%) tuberculosis related serpiginous patients fulfilled the inclusion criteria. Mean age was 39 ± 5.3 years. Snellen best corrected visual acuity (BCVA) at presentation was 0.96 ± 0.08, excluding the patient with bilateral macular involvement. Mean follow up was 87.8 ± 63.2 months. In 3/4 patients, treatment with multiple tuberculostatic therapy combined with multiple immunosuppressive agents, started at presentation, within 4, 8 and 66 months after first signs of disease onset, was shown to stop the progression of the disease, with a retained visual acuity of 1.0. One patient had bilateral macular involvement before being seen in our center, and bilateral visual acuity was less than 0.1 in both eyes. After initiation of dual combined treatment with multiple anti-tubercular and multiple immunosuppressive therapy visual acuity improved to 0.25 OD (oculus dexter) and 0,05 OS (oculus sinister) and presented substantial visual field improvement (see case N°4).

### Cases

#### Case 1

A 37-year-old man was referred to our centre for a second opinion for a bilateral choroiditis. He had been diagnosed with bilateral choroiditis 2 years earlier and had received an undetermined course of systemic prednisone treatment. Four months before we saw the patient, he was taken care elsewhere for bilateral optic disc swelling and reactivation of choroiditis. During the investigations of the choroiditis a positive T-spot-TB test was found but the infectiologists excluded an active TB. The patient was first treated with a high dose of oral prednisone (100 mg per day). After six weeks the optic disc swelling grew, and the choroidal lesions increased in size and number. Prednisone therapy was lowered and stopped. In parallel, a quadruple anti-TB therapy was installed without any effect on the papillitis, progression of choroidal lesions and an increase of inflammation. The patient was referred to our centre for further management.

**Fig. 1:**
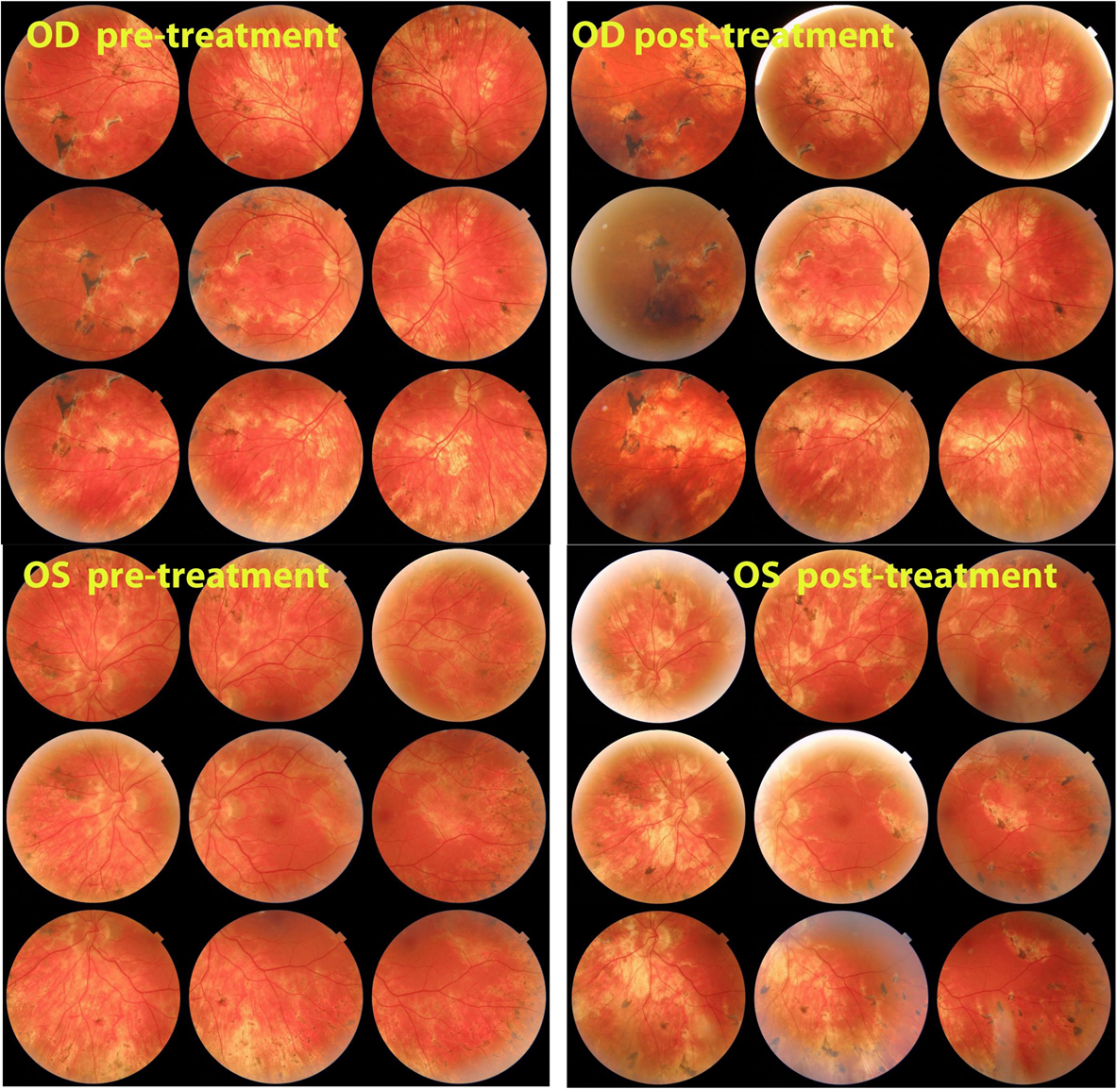
Patient 1 OD (top) Fundus panorama pictures at presentation pre-treatment and at the end of follow-up post-treatment Patient 1 OS (bottom). Fundus panorama at presentation pre-treatment and at the end of follow-up post-treatment

At presentation the patient had a BCVA of 1.25 OU. There was no anterior inflammation at the slit-lamp with, however, subclinical inflammation measured with LFP, amounting to 8.9 ph/ms OD and 11.0 ph/ms OS (normal values 3–6 ph/ms). Vitreous examination revealed rare vitreous cells OD and 1+ vitreous cells OS. Fundus examination revealed serpiginoïd lesions all over the mid-periphery in both eyes, sparing both foveas (Fig. 1).

Visual fields demonstrated scattered scotomas corresponding to the choroiditis lesions within the posterior pole that resolved until the end of follow-up**.** Microperimetry showed a decrease of retinal sensitivity OS>OD, showing that there was even a decrease of retinal sensitivity in central areas where there were no areas of chorioretinitis with substantial increase of the microperimetry score towards normal sensitivity until the end of follow-up.

FA showed scattered areas of hyperfluorescence representing window-effect due to the loss of the RPE screen. FA signs did not change substantially during follow-up and this imaging modality was not useful to monitor disease evolution.

**Fig. 2:**
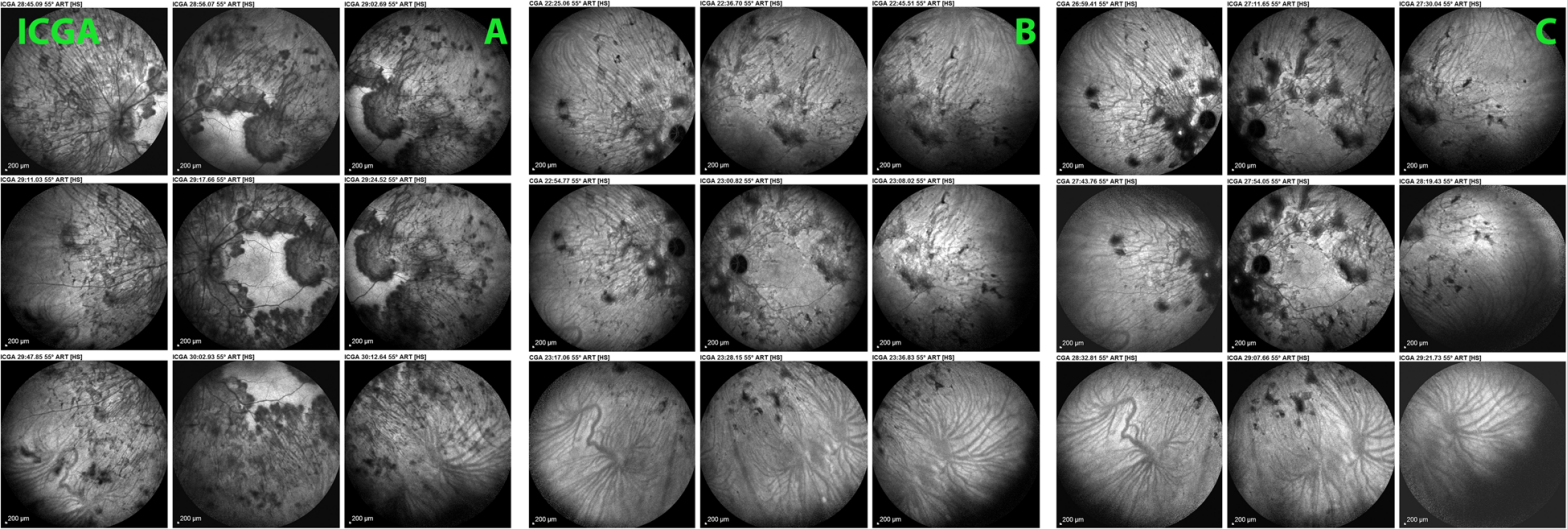
Patient 1 OS. Evolution of ICGA (intermediate angiographic phase). At presentation (A), widespread hypofluorescent, dark areas all over the fundus, indicating either chorioretinal atrophy or inflammatory choriocapillaris non perfusion; preservation of the central macula. Nine months after initiation of treatment (B), widespread areas over the whole fundus regain normal ICGA fluorescence indicating recovery of choriocapillaris perfusion; the remaining hypofluorescent areas correspond to chorioretinal atrophic areas. Three years after initiation of therapy (C), further slight recovery of choriocapillaris perfusion (normal ICGA fluorescence) and slight enlargement of the chorioretinal atrophic areas seen on B

ICGA revealed extended areas of hypofluorescence at presentation with only the central macula showing normal fluorescence. Areas of hypofluorescence indicated either chorioretinal atrophy or choriocapillaris non perfusion. The latter areas of choriocapillaris non perfusion progressively regained normal fluorescence with time under treatment, while areas of complete chorioretinal atrophy at presentation remained hypofluorescent representing scarred areas (Fig. 2).

**Fig. 3:**
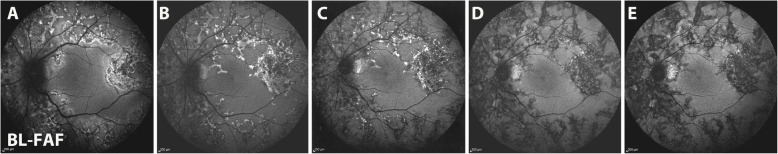
Patient 1 OS. Evolution of blue light fundus autofluorescence (BL-FAF). At presentation (A), numerous lines of BL-FAF bright hyperautofluorescence due to accumulation of lipofuscin products from progressing destruction of photoreceptors. Seven weeks later (B), slight decrease of bright hyperautofluorescent rims but apparition of newly affected areas. Four months later (C), substantial decrease of bright hyperautofluorescence at the border of lesions. One year later (D), hyperautofluorescent rims have almost completely disappeared indicating complete arrest of progression of lesions, confirmed 6 months later (E)

BL-FAF showed bright hyperautofluorescent rims at the level of progressing lesions around the preserved macular region produced by accumulation of lipofuscin products generated by inflammatory involvement of new fundus areas (Fig. 3). After introduction of dual multiple anti-tubercular and multiple immunosuppressive therapy the bright hyperautofluorescent rims faded and disappeared (Fig. 3).

SD-OCT was normal at the level of the macula but showed RPE-photoreceptor atrophy on the portions of sections through the lesions OU.

**Fig. 4:**
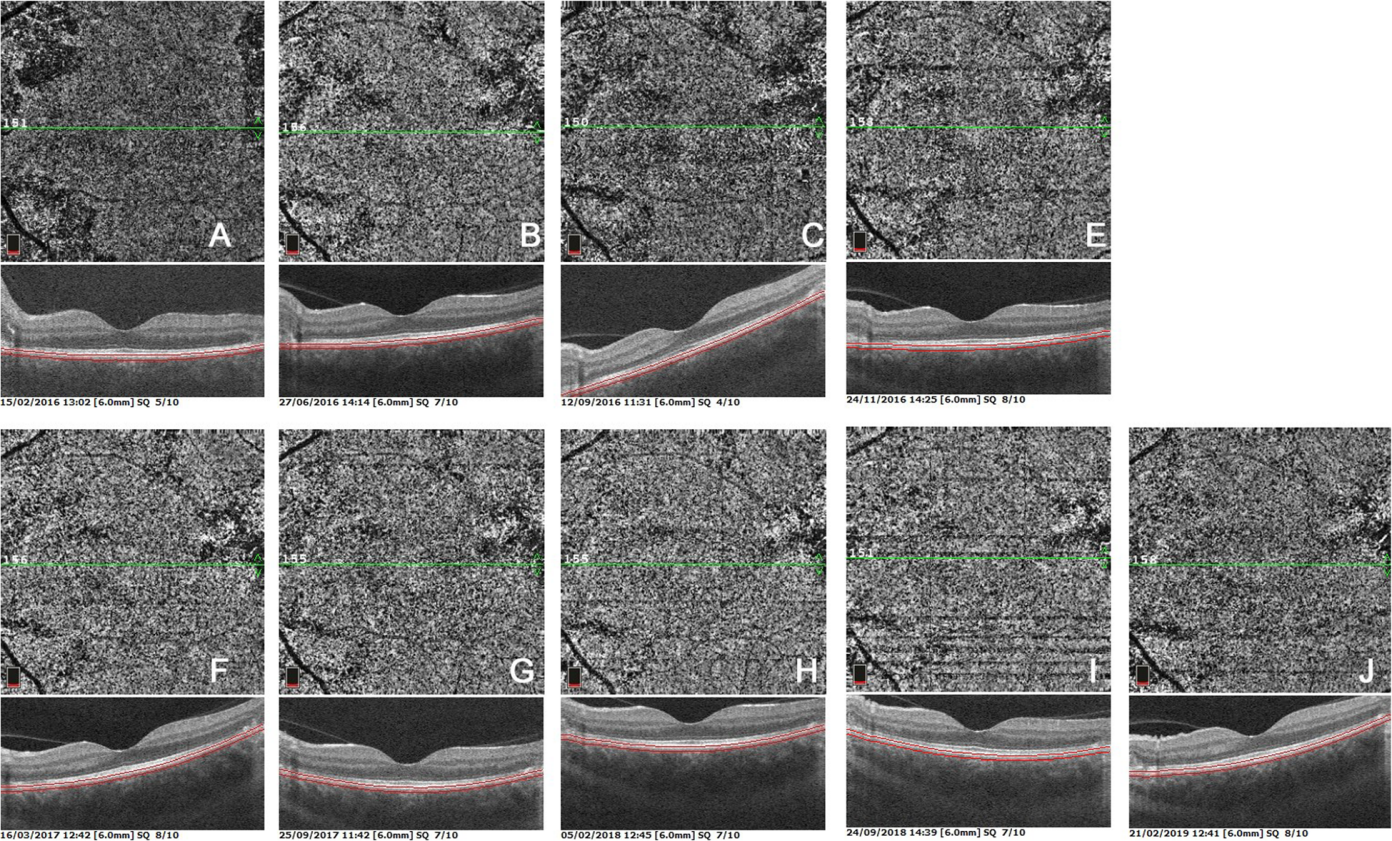
Patient 1 OS. Evolution of OCT-angiography (OCTA). OCTA frames at presentation (A), showing several areas of choriocapillaris non perfusion with some new lesions visible at 4 and 7 months (B, C) and slight decrease of lesions seen at 9 (E), 12 (F), 18 (G), 24 (H), 30 (I) and 36 months (J). OCT-A was not very precise to follow lesions as the central macula was mostly preserved but also because the delineation of lesions did not show clear borders

OCT-A also showed reduction of the few drop-out areas seen at the border of the central conserved areas (Fig. 4). This imaging modality was however less demonstrative compared to ICGA, as most of the lesions were beyond the macula in the mid-periphery and periphery (Fig. 2).

Initial treatment after the first visit consisted of quadruple anti-tubercular therapy (Isoniazid, rifampicin, ethambutol, pyrazinamide), prednisone (80 mg/day), cyclosporine (4.8 mg/kg/day) and infliximab (5 mg/kg every 4 weeks).

Anti-tubercular therapy was discontinued after 15 months, and multiple immunosuppressive therapy was tapered and discontinued after 36 months. Recurrence-free follow-up was 36 months (Table 1).

**Table 1:**
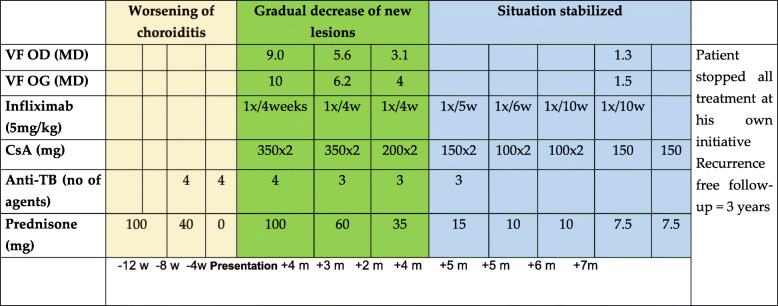
Schematic representation of the course of treatments in patient 1

*VF* visual field, *MD* mean deviation, *CsA* Cyclosporin, *w* weeks, *m* months, *Anti-TB* anti-Tuberculosis treatment

#### Case 2

**Fig. 5:**
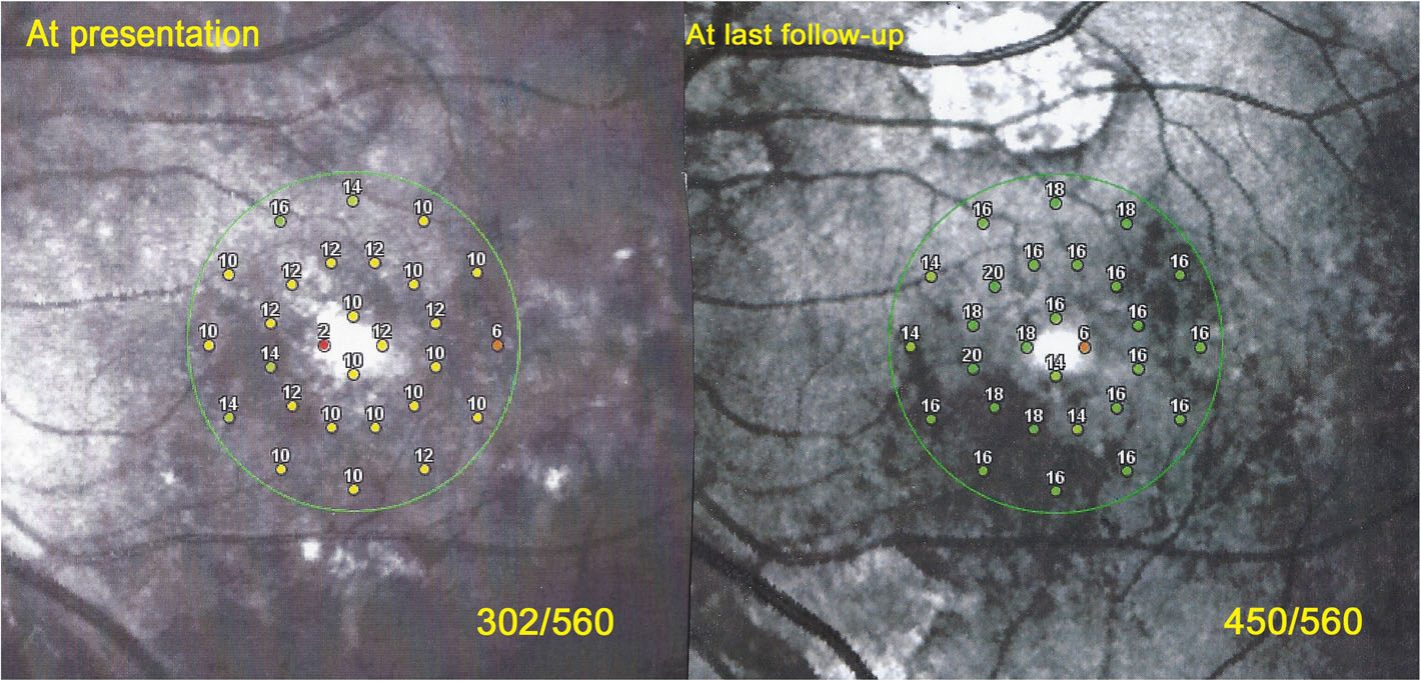
Patient 2. Evolution of microperimetry OS at presentation till last follow-up. Microperimetry at presentation (left picture) shows decreased retinal sensitivity to a score of 302/560. After treatment the score increased to 450/560 (right picture)

**Fig. 6:**
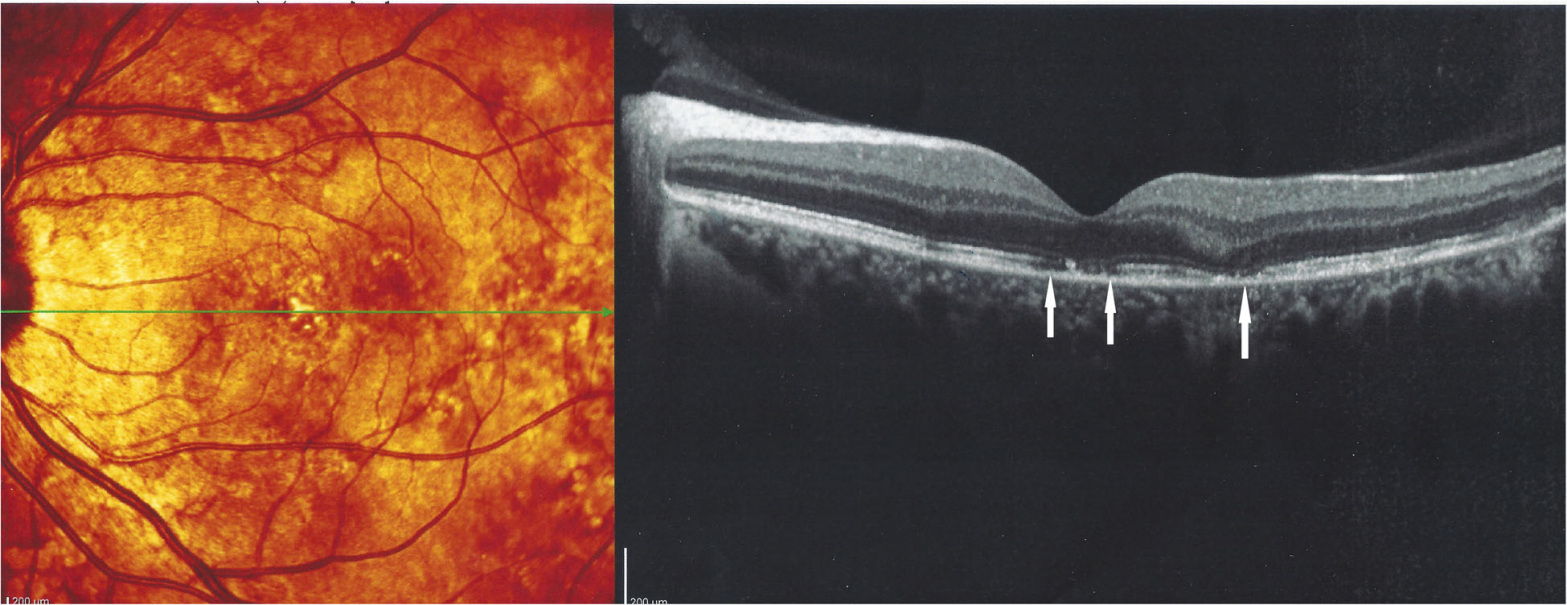
Patient 2. SD-OCT of patient 2. Shows inhomogeneity on fundus scanning laser photography OS corresponding to areas of photoreceptor loss (white arrows)

**Fig. 7:**
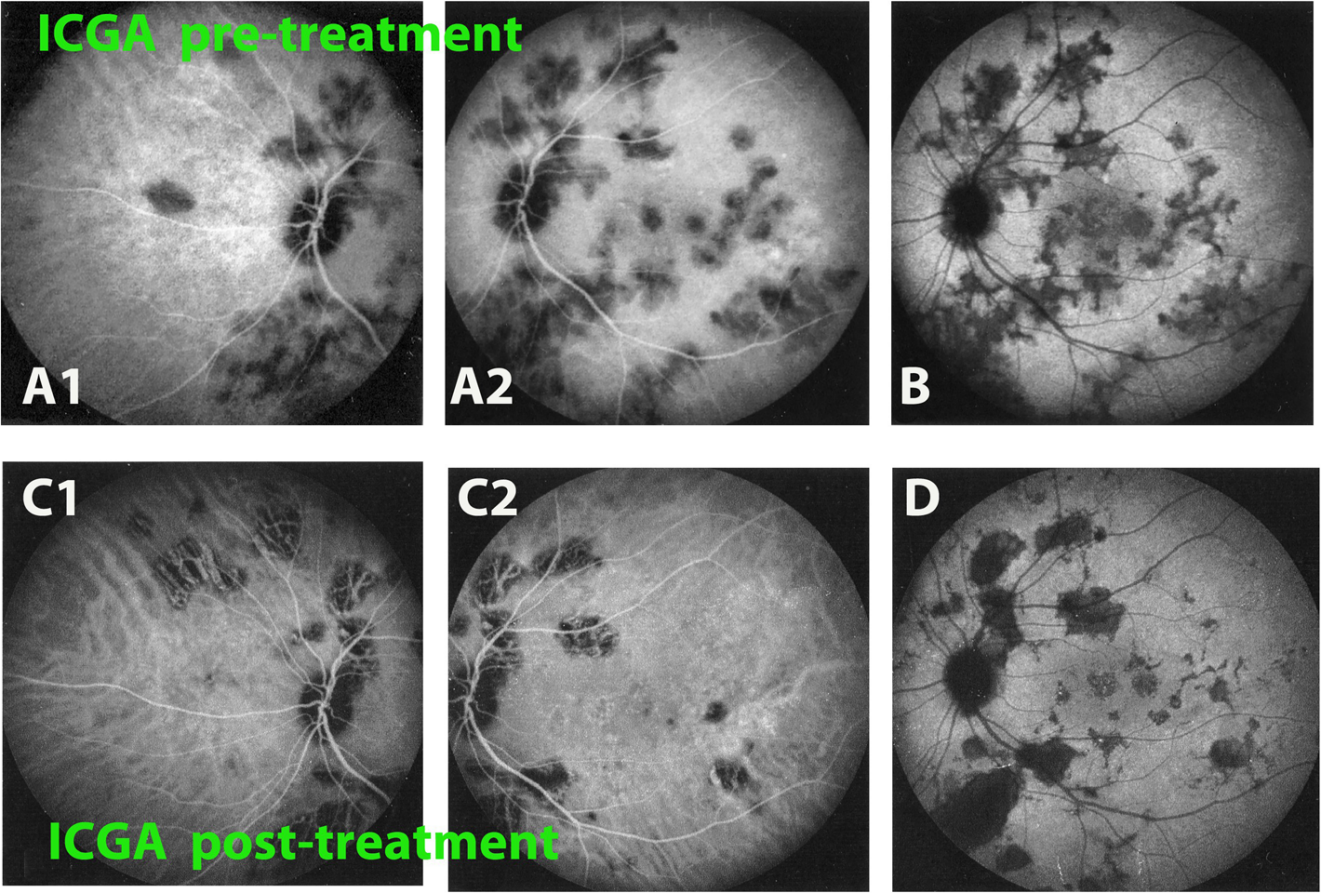
Evolution of ICGA of OS in patient 2 at presentation and last follow-up. ICGA of OS at presentation showed hypoperfusion in the intermediate (A1 & A2) and the late phase of angiography (B) due to choriocapillaris non perfusion and/or scaring. At the end of follow-up, decreased number of hypofluorescent areas in the intermediate (C1 & C2) and late phase of angiography (D). The remaining hypofluorescent areas correspond to irreversible chorioretinal atrophy

A 42-year-old woman was referred to our center for a choroiditis of her left eye which had been treated with high dose systemic corticosteroid therapy during 4 months without notable effect. At presentation she was still under low oral prednisone (7.5/5 mg/on alternate days) BCVA was 1.0 in her right eye and 0.8–0.9 in the left eye. The anterior segment showed no inflammation on slit lamp examination and there were no vitreous cells ODS. Intraocular pressure (IOP) was normal ODS (12–13 mmHg). Fundus examination of the right eye was normal. The left fundus showed multiple yellow-white placoid multifocal lesions in the posterior pole and mid-periphery. Functional tests and multimodal imaging of the right eye were normal. The left visual field showed several small scotomas with a mean defect (MD) value of 4.4. Left microperimetry showed a decreased central retinal sensitivity to 302/560, which increased to 450/560 at the end of the follow-up, **(**Fig. 5**)** explained by photoreceptor damage and subsequent recovery on SD-OCT **(**Fig. 6**)**. FA showed hyperfluorescent areas corresponding to window effects of diseased areas. The most useful imaging modality was ICGA **(**Fig. 7**)**, clearly showing all the areas of choriocapillaris non perfusion until the late angiographic stages. ICGA showed many more areas than FA. An IGRA test (QuantiFERON-TB™) was clearly positive. The diagnosis of unilateral tuberculosis related multifocal-serpiginous choroiditis was posed. We started an anti-tubercular tri-therapy, increased to quadri-therapy after 3 weeks (ethambutol, isoniazid, rifampicin and pyrazinamide) together with oral prednisone (40 mg/day representing 1 mg/kg) and azathioprine followed by mycophenolic acid (Myfortic® 720 mg twice daily) because of azathioprine intolerance. Erythema nodosum appeared after the initiation of antitubercular therapy that was treated by her family doctor.

Antitubercular therapy lasted for 15 months, and the total length of treatment was 24 months. Recurrence-free follow-up without treatment was 9 years.

#### Case 3 (brief summary)

**Fig. 8:**
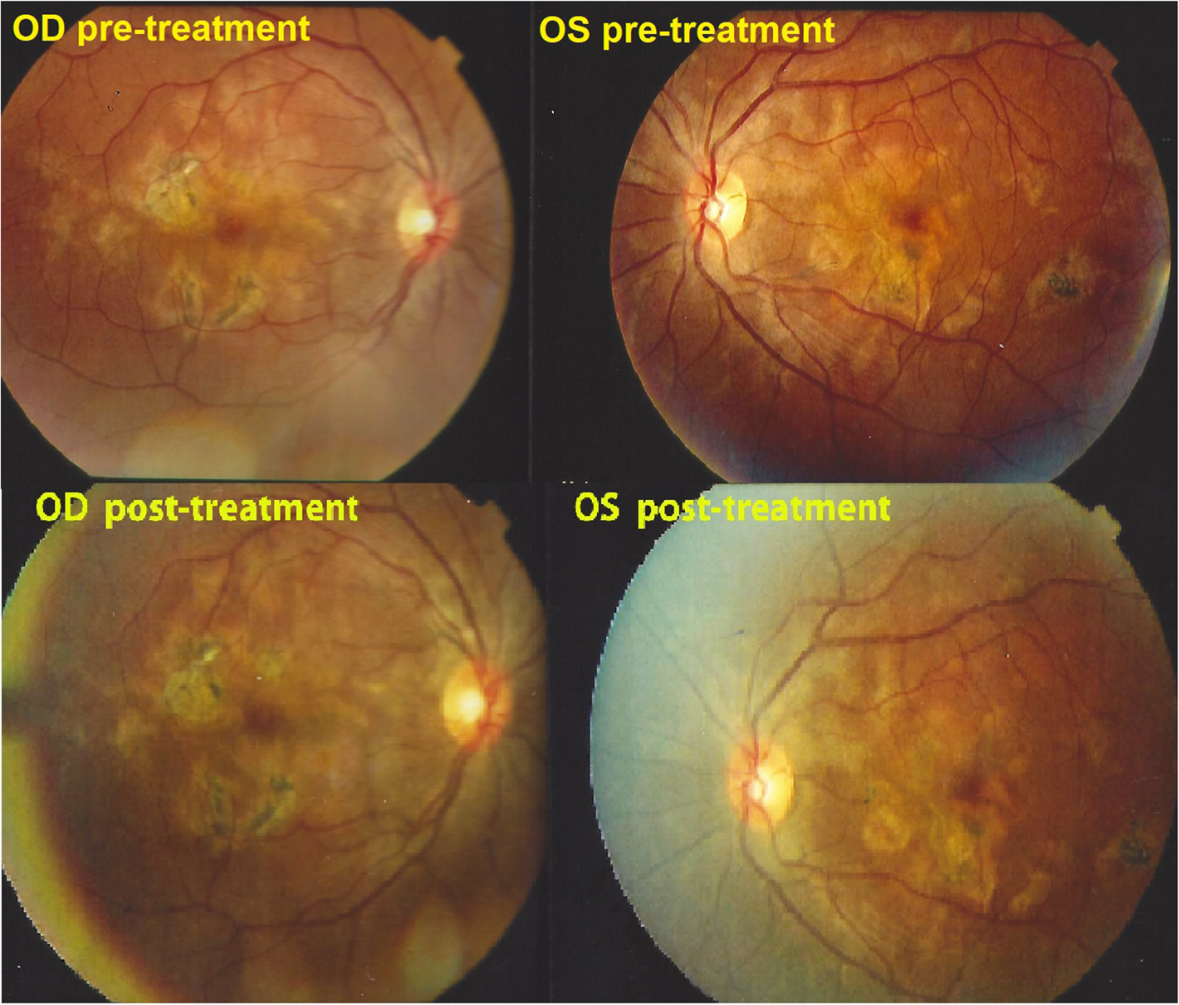
Patient 3. (top) Fundus ODS at presentation. Numerous serpiginoïd and placoid lesions. (bottom) Fundus ODS at last follow-up visit. No additional lesions noted, although the exact comparison is difficult. Lesions are more pigmented

**Fig. 9:**
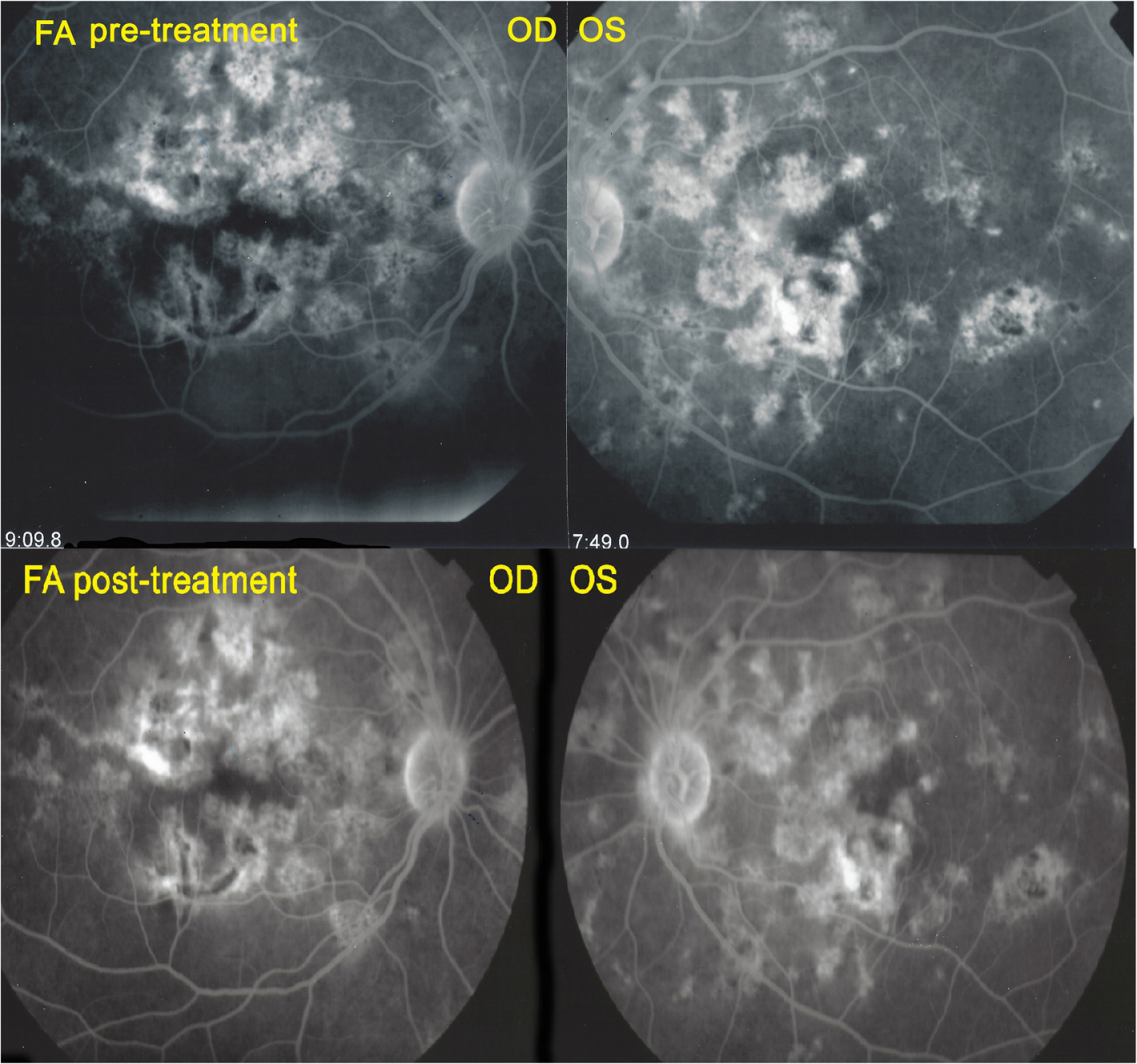
Patient 3 (top) FA at presentation. Numerous hypofluorescent areas explained by window effect. (bottom) FA at final follow-up. There seems to be no evolution of the lesions. However, FA is of marginal use to follow-up serpiginous lesions

**Fig. 10:**
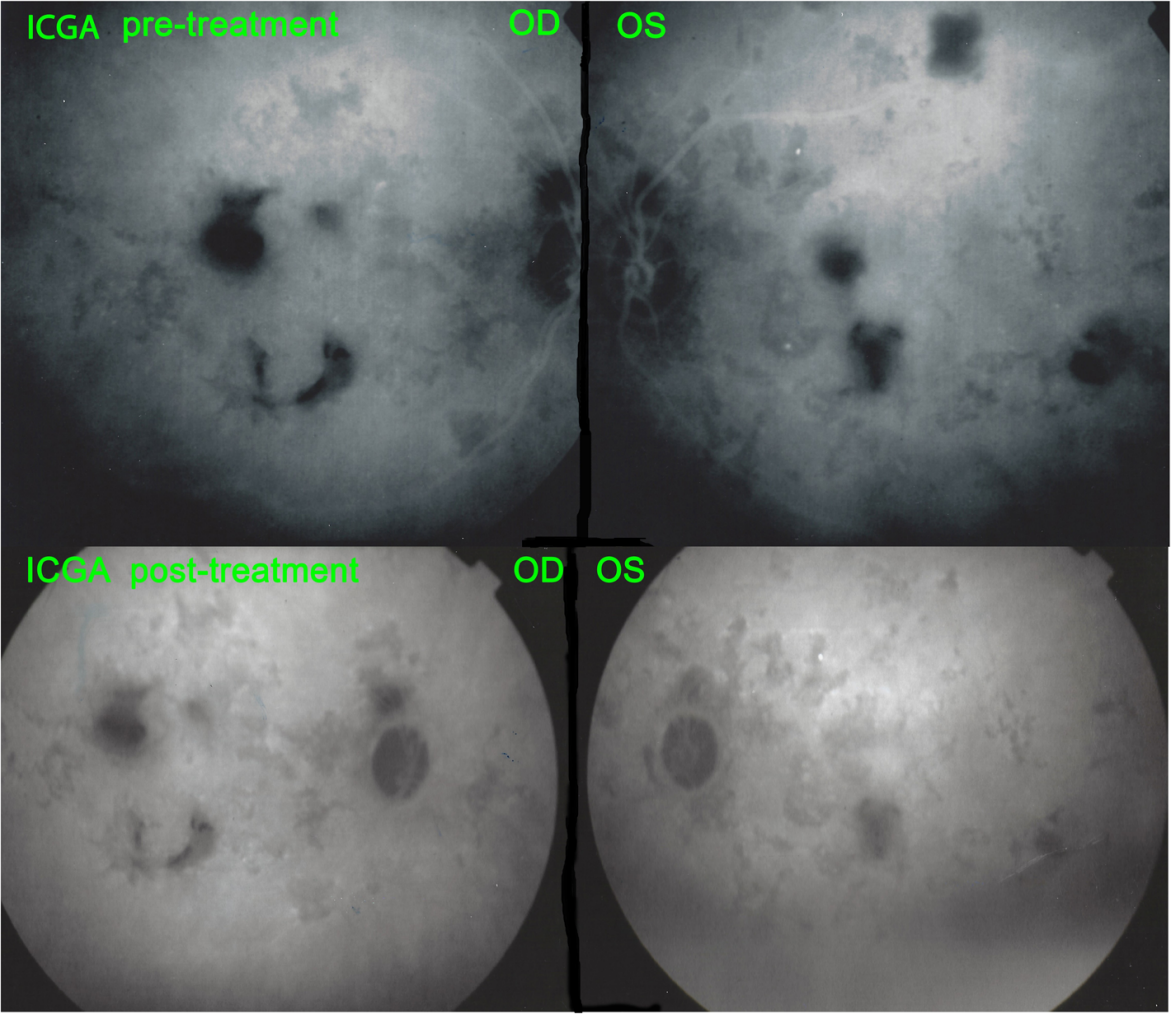
Patient 3 (top) ICGA at presentation. Hypofluorescent areas correspond to choriocapillaris non perfusion or to chorioretinal atrophy. (bottom) ICGA at last follow-up. Slight decrease of number of hypofluorescent areas corresponding to choriocapillaris re-perfusion. The remaining hypofluorescent lesions correspond to chorioretinal atrophic scars

A 32-year-old Indian lady had been diagnosed with bilateral serpiginous choroiditis 3 years before she consulted our centre and was treated with a course of systemic corticosteroids for two months. Two years before consulting, the chorioretinitis had been diagnosed as of tubercular origin but the patient first declined anti-tubercular therapy. When we first saw the patient, treatment was still not accepted despite progression of disease (Fig. 8). When dual multiple antitubercular and multiple immunosuppressive was introduced 66 months atter the first signs, the situation stabilised (Figs. 8, 9, 10). Duration of anti-tubercular therapy was 12 months. Total treatment duration was 16 months and recurrence free follow-up was 2 months, after which the patient was lost to follow-up.

#### Case 4 (brief summary)

**Fig. 11:**
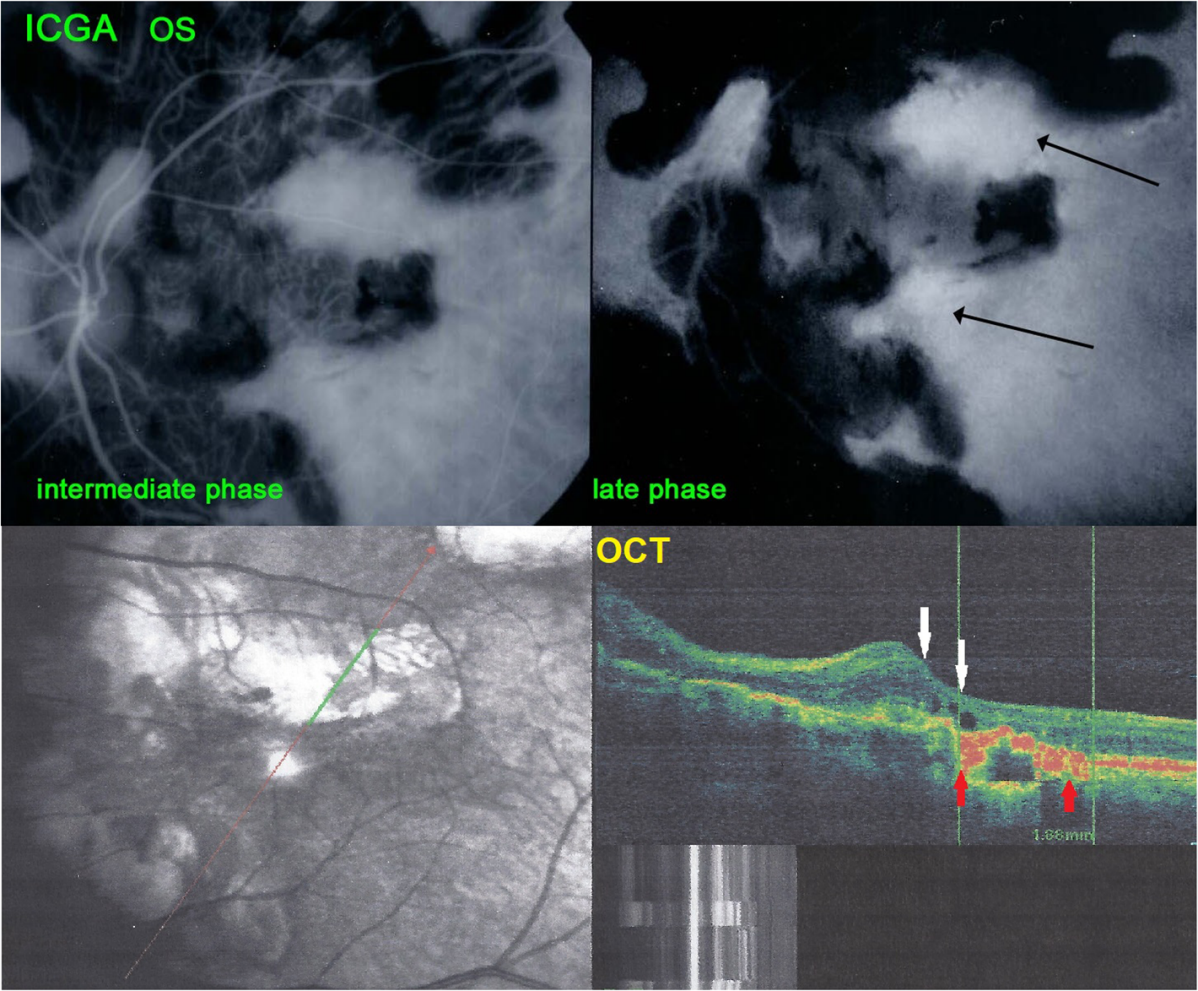
Patient 4 (top) ICGA hyperfluorescence indicating progression of choriocapillaritis. The left frame shows the posterior pole of the left eye with chorioretinal scars (hypofluorescent areas) in the intermediate angiographic phase. The right picture shows the same area in the late angiographic phase characterized by hyperfluorescence between scarred areas indicating progression of choriocapillaritis (black arrows). (bottom) SD-OCT showing oedema (white arrows) at the border of cicatricial areas (red arrows). These edematous areas are susceptible to be recovered after introduction of dual antitubercular and immunosuppressive therapy explaining the improvement of the visual fields shown in Fig. 21

**Fig. 12:**
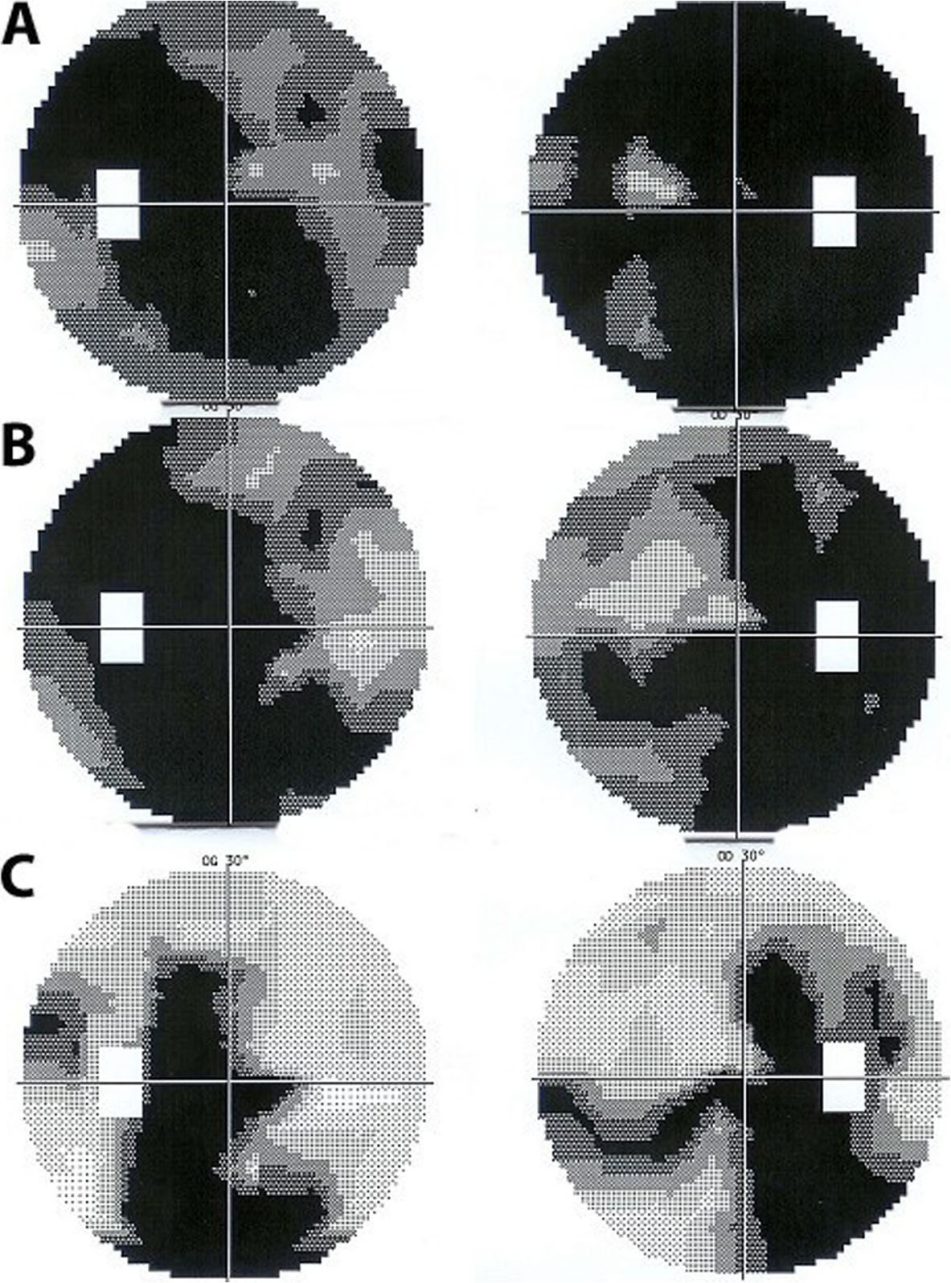
Patient 4. Evolution of visual fields ODS after introduction of dual anti-tubercular and immunosuppressive therapy. Visual fields at presentation (top pair of images) (A), improved progressively after introduction of dual anti-tubercular and immunosuppressive therapy (B & C). The areas recovered most probably correspond to the healed edematous areas shown on Fig. 20

A 44-year-old man was referred because of progressing SC for the last 11 years. He had been treated several times with systemic prednisone and other immunosuppressive agents that was unable to stop the evolution. When we saw the patient for the first time, his visual acuity was hand movements ODS and widespread SC included both maculas. ICGA showed hyperfluorescent areas adjacent to chorioretinal scars indicating inflammatory activity (Fig. 11). These ICGA hyperfluorescent areas corresponded to oedematous retina bordering the chorioretinal scars (Fig. 11). IGRA testing (Quantiferon-TB) was strongly positive. Multiple antitubercular therapy was initiated, starting with a quadri-therapy. In parallel systemic prednisone, mycophenolate acid therapy (Myfortic®) and cyclosporine were added. Visual acuity improved slightly to 0.25 OD and 0.05 OS. Visual field also improved substantially (Fig. 12), probably due to the reversibility of the oedematous lesions at the border of SC scars (Fig. 11). Duration of anti-tubercular therapy was 24 months and immunosuppressive therapy at reduced dosages (Cyclosporine and Myfortic®) was maintained during the following 15 years, as the patient signaled worsening each time discontinuation was attempted.

### Summary of findings

Data on patients are summarized on Table 2. The time lag until dual multiple anti-tubercular and immunosuppressive therapy was started was quite long, from 4 months to eleven years (mean 52.5 ± 60 months), explaining the advanced stage of lesions. The mean total duration of treatment was 25.3 ± 10 months, when excluding patient 4 still under treatment. The mean duration of antitubercular treatment was 16.5 ± 5.2 months. In two patients a prolonged follow-up was achieved with a recurrence free follow-up of 30 months and 9 years respectively (mean 69 ± 55.1 months).

**Table 2:** Patients, treatment data, visual acuities, visual fields and follow-up

Patients	Gender/Age	Eye OD/OS	Time to 1st visit	Duration of Anti-TB ttt	Total duration of treatment	Recurrence free, without treatment	VA 1	VA 2	VF 1 (MD)	VF 2 (MD)
Patient 1	M/38	OD	4 months	15 months	36 months	36 months	1.0	1.0	9	1.3
		OS					1.0	1.0	10	1.5
Patient 2	W/42	OD	8 months	15 months	24 months	9 years	1.0	1.0	2.8	2.1
		OS					0.9	1.0	5.3	5.0
Patient 3	W/32	OD	66 months	12 months	16 months	2 months (Lost to F-UP)	1.0	1.0	−1.4	−1.3
		OS					10	1.0	−1.1	−1.6
Patient 4	M/44	OD	12 years (132 m)	24 months	Still under treatment (15 years)	Still under treatment	0.1	0.16	25.3	15.6
		OS					0.05	0.16	19.4	16.6

*Ttt* treatment, *VA* visual acuity, *VF* visual field, *MD* mean deviation, 1 = at presentation, 2 = last visit

Visual acuity remained full in all 3 patients who presented before the fovea was involved and for whom treatment could be stopped. Octopus® Visual fields improved significantly in patients having visual field defects at presentation with a decrease of mean defect (MD) from 12.06 ± 8.6 to 7.05 ± 7.14 (*p* < 0.03). In the two patients for whom microperimetry was available, it increased substantially in the eyes where it was decreased at presentation, from 358/560 to 556/560 OS in patient 1 (Fig. 5) and from 302/560 to 450/560 OS in patient 2 (Fig. 10).

## Discussion

Serpiginous-like Choroiditis is an immune-mediated choriocapillaritis related to *Mycobacterium tuberculosis*. The RPE has been shown to harbour *Mycobacterium tuberculosis*, a site difficult to reach for anti-tubercular therapy [15]. Therefore, prolonged anti-tubercular therapy is needed but does not address the fundamental immune mechanism at the origin of the disease and aggressive immunosuppressive therapy seems to be needed in parallel. Before receiving concomitant multiple anti-tuberculous therapy and multiple immunosuppression, all 4 patients reported here had progressed either because the two treatments were not given together or with insufficient dosages or only immunosuppressive treatment was given. The progression of the disease was stabilized and later stopped by aggressive dual multiple anti-tubercular and multiple immunosuppressive therapy. In three patients the therapy could be stopped, and they had a treatment free mean follow-up of 69 months. The last patient seen after 11 years of treatment limited to immunosuppression without anti-tubercular treatment had lost most of his central vision. When dual multiple antitubercular and immunosuppressive therapy was started, after a positive IGRA test was received, he improved his visual fields substantially. However, treatment could never be stopped entirely, as worsening occurred each time discontinuation was attempted.

There is no consensus on the proper management of SC. Many immunosuppressives with different dosages and different combinations have been reported [16–19]. Majunder et al., in their extensive review on SC, gave a detailed account on treatments used in SC [1]. In the literature, a trend was found towards treatments which may have severe life-threatening side-effects such as chlorambucil [20–22] and cyclophosphamide [23, 24], an indication on how difficult and how unsatisfactory the treatment of this condition appears to be. This was a stimulus for us to opt for an immunosuppressive therapy with multiple agents. This approach has been reported in two studies which used triple immunosuppression with positive results in 6 patients [25, 26]. Unlike any other uveitis resistant to classical immunosuppressants, biologicals have to be used with caution in tuberculosis-related SC as tuberculosis can be reactivated with fatal results [1, 27].

The positive point, nowadays, is that we can follow the progression of the disease with more precision using ICGA, which also allows us to verify whether a given treatment or treatment combination proves efficient or not. In our hands, aggressive therapy with multiple immunosuppressive agents was always necessary to halt the progression of the disease [11].

In IGRA-positive serpiginous patients, aggressive immunosuppression is not sufficient as the immune-mediated process keeps stimulating choriocapillaris inflammation. Aggressive anti-tubercular therapy is necessary as a complement.

This is in accordance with studies in endemic areas from Gupta et al., which showed that simultaneous treatment with Anti-TB and corticosteroids is necessary and is a better therapeutic option rather than anti-tubercular therapy alone [1]. Considering the difficult access of anti-tubercular treatments to the RPE reservoir of buried mycobacteria, we maintained anti-tubercular therapy for longer than usual [15].

As seen in our first case, the initiation of the anti-tubercular treatment elsewhere provoked a paradoxical Jarish-Herxheimer type worsening of inflammation, as has been described by several authors [28, 1]. In patient 2, a Jarish-Herxheimer type of immunologic reaction occurred with the apparition of an erythema nodosum. Ganesh et al. have evaluated the available literature on paradoxical reactions/worsening in TB in their review and described paradoxical worsening of ocular lesions after initiating an anti-tubercular treatment of ocular tuberculosis including serpiginous-like choroiditis [29].

Progression of the disease and unfavorable outcome of IGRA-positive serpiginous choroiditis occur when both anti-tubercular and immunosuppressive treatments are not given concomitantly and/or in an insufficiently aggressive manner which is what happened in our 4 patients before the approach we describe was initiated. The fact possibly at the origin of a too timid immunosuppression is the fear to give an aggressive immunosuppressive therapy in IGRA positive patients.

In conclusion, serpiginous like choroiditis is a rare immune-mediated condition triggered by tuberculosis with deleterious complications for the eye. Early, aggressive treatment with multiple anti-TB antibiotics and multiple immunosuppressive agents can stop the evolution and preserve vision.

## Data Availability

Please refer to corresponding author.

## Abbreviations

IGRA: Interferon Gamma Release Assays
SD-OCT: Spectral-Domain Optic coherence tomography
BCVA: Best corrected vision acuity
SC: Serpiginous choroiditis
RPE: Retinal pigment epithelium
TB: Tuberculosis
EDI-OCT: Enhanced depth imaging OCT
OCT-A: Optical coherence tomography angiography
BL-FAF: Blue light fundus autofluorescence
FA: Fluorescein angiography
ICGA: Indocyanine angiography
IOP: Intraocular pressure
LFP: Laser flare photometry
OD: Oculus dexter
OS: Oculus sinister
